# Highly educated patients have lower dental compliance during the COVID-19 pandemic: an observational study

**DOI:** 10.1186/s12903-022-02307-x

**Published:** 2022-07-12

**Authors:** Yu-Hsiang Chou, Ying-Chu Lin, Mei-Hsuan Lee, Yu-Ting Huang, Pei-Feng Liu, Chung-Lin Huang, Kai-Fang Hu

**Affiliations:** 1 School of Dentistry, College of Dental Medicine, Kaohsiung Medical University, Kaohsiung, Taiwan; 2 Division of Periodontics, Department of Dentistry, Kaohsiung Medical University Hospital, No.100, Tzyou 1st Road, Kaohsiung, 807 Taiwan; 3 Institute of Clinical Medicine, National Yang Ming Chiao Tung University, Taipei, Taiwan; 4 Division of Medical Statistics and Bioinformatics, Kaohsiung Medical University Hospital, Kaohsiung Medical University, Kaohsiung, Taiwan; 5 Department of Medical Research, Kaohsiung Medical University Hospital, Kaohsiung Medical University, Kaohsiung, Taiwan; 6 Department of Biomedical Science and Environmental Biology, Kaohsiung Medical University, Kaohsiung, Taiwan; 7 Department of Medical Research, Kaohsiung Medical University Hospital, Kaohsiung, Taiwan; 8 Center for Cancer Research, Kaohsiung Medical University, Kaohsiung, Taiwan; 9 Institute of Biomedical Sciences, National Sun Yat-Sen University, Kaohsiung, Taiwan

**Keywords:** COVID-19, Dental compliance, Dental appointment, Attendance

## Abstract

**Background:**

The outbreak of coronavirus disease 2019 (COVID-19) is rapidly changed medical habits, and dental clinics have been forced to adapt. This study explored the pandemic-induced changes in patient utilization of dental services to assist practitioners in responding efficiently to similar public crises as references in the future.

**Methods:**

We retrospectively analyzed the correlation between patient profiles and dental visits attendance within 2 months before and during the outbreak.

**Results:**

A total of 332 patients, 210 women and 122 men (total number of visits: 1068) were enrolled in this study. A significantly lower attendance rate was noted during the COVID-19 period (70.3%) than prior to the pandemic (83.4%). The rate of return visits for patients with a high education level during the COVID-19 period was significantly reduced from 96.5 to 93.1%. In addition, the number of days between two visits significantly increased during the pandemic.

**Conclusions:**

Our results indicate that, during the pandemic period, the attendance rates of return dental appointments decreased, and the rate of missed appointments for patients with a high educational levels was higher than that of patients with a low educational level.

**Clinical relevance:**

Preventive management of these patients who are easy to miss dental appointments may enable more effective use of medical resources.

## Background

The 2019 outbreak of a novel respiratory virus, SARS-CoV-2, resulted in an ongoing global pandemic, COVID-19. COVID-19 spread rapidly across the globe after its initial cases were reported in China in December 2019. The COVID-19 epidemic, originating in Wuhan, China [1], became a major public health challenge not only for China but also countries around the world.

The number of confirmed cases in Taiwan was 283 in March 2020. Since late January 2020, the governments of various countries including Taiwan have recommended that people avoid gathering in crowded places and maintain proper social distance to prevent the rapid spread of the SARS-CoV-2 virus. The fear of COVID-19 infection and its rapid transmission have resulted in less frequent visits to crowded places, such as dental clinic and hospital. The fear is not only because they are crowded places, but also because they are healthy places. Considering specifically dental clinics the level of infection can be very high since you will have to take off your mask, dentist will work with aerosol, etc. Economic issues can also be a problem to go to a dentist during the pandemic.

A main transmission route of COVID-19 is droplet infection, including infection through inhalation or mucosa contact with patient’s blood, saliva, and other body fluids [2]. Dental treatment procedures are considered to pose a high risk of spreading COVID-19 [3–5], especially those incorporating the use or ultrasonic device that can produce aerosol release. The health authorities of some cities have ordered dental institutions to suspend nonemergency dental services. In many places, dentists have not been permitted to provide conventional dental treatment, being instead restricted to only handling urgencies and emergencies. The government policies and personal considerations have caused considerable anxiety and confusion among patients, and many of them may have altered their utilization of dental service, medical compliance, and intention to attend dental appointments. COVID-19 has affected the delivery of vital health care services for many patients indicating that they would not visit only in an emergency [6].

Many treatment guidelines recommend that dental treatments shall be performed in personal protective equipment with the appropriate protective measures taken [7–9]. As the pandemic continues, offending dental treatment only in emergencies will become infeasible. When the risk of disease transmission was high, the efficient use and organization of medical staff and resources were essential. Dental clinics are largely functioned and widely used through an appointment system. If a patient schedules an appointment but fails to attend, resources and the time of medical personnel are wasted. Understand the patients’ intend to attend dental appointments and avoiding the waste of medical resources can lead to greater efficiency in dental clinic treatment during pandemic condition.

A 2013 study on medication adherence noted that patients with higher education levels had better adherence to medication use [10]. The 2021 study found that regular dental visit attendance had better compliance among women and those with higher education [11]. However, research on dental appointment during the COVID-19 pandemic is rare. The aim of present study explored the pandemic-induced changes in patient utilization of dental services to assist practitioners in responding efficiently to similar public crises as references in the future.

## Methods

In this study, a retrospective analysis was conducted using the data of patients receiving outpatient dental clinical services at a Medical Center hospital in Kaohsiung, Taiwan. The study period of during -COVID-19 pandemic was from February 1 to March 31, 2020. The pre-COVID-19 control period was between October 1 and November 30, 2019, prior to any policies or public concern. The patients analyzed in this study were seen by the same attending physician. All patients were jointly cared for by attending physicians, residents and dental hygienists. The patients’ demographic characteristics were educational level, age, and gender as well as attendance, and interval between different appointments were recorded. Educational levels were classified into two groups: low education level (high school or lower, ≦12 years) and high education level (university and post-graduation or higher, > 12 years). Age was grouping as ≧ 50 years old and < 50 years old. Pre-COVID-19 appointment intervals were recorded the numbers of days between the return visit and the previous visit. During COVID-19 appointment intervals were recorded the numbers of days between the return visit and the next visit during COVID-19 pandemic. Appointments were made by patients and medical personnel for return visits and were recorded on the electronic appointment system. Our data collection was backtracked by the electronic appointment system and electronic medical records. All of the data was collected by one author and checked for completeness by another author.

The study protocol was approved by the Institutional Review Board of the Kaohsiung Medical University Hospital, Taiwan (KMUHIRB-E(I)-20,210,307).

Continuous variables are presented as mean ± standard error (SE) and analyzed through two-sample t-test. Categorical variables are presented as number and percentage and were analyzed through using Chi-square test. The results were accompanied by statistical analysis that yielded an odds ratio and 95% confidence interval using multiple logistic regression in JMP statistical software (SAS, Cary, NC, USA). The significance level was set at p < 0.05.

## Results

A total of 332 patients were included in this study, including 210 women and 122 men. A total of 261 people were seen during the Pre-COVID period, and the number of visits was 550. On the other hand, a total of 254 people were seen during the COVID period, with the number of visits was 518. The analysis was based on the number of visits. A total number of visits was 1068 enrolled in the present study. Calculated by the number of visits, their demographic characteristics are listed in Table 1. No significant difference was noted in the gender distribution of the patients during these two periods. The mean age was 53.01 ± 0.51 and 49.57 ± 0.52 years for the participants from the pre-COVID-19 and During COVID-19 periods, respectively. The age of pre-COVID-19 patients were significantly older (p < 0.0001). No significant difference in the distribution of patients’ educational levels was observed between two study groups. The attendance rate was fell significantly from 83.4% before the pandemic to 70.3% during the pandemic (p < 0.0001). Further to compare the characteristics of the patients who attended dentists or missed treatment during these two periods (Table 2). The rate of appointment absenteeism during the COVID-19 period (29.7%) was significantly higher than that during the pre-COVID-19 period (16.6%; p < 0.0001). However, the differences in the distributions of age and gender did not differ significantly.

**Table 1:** Characteristics of patients who used dental service before and during the COVID-19 pandemic (n = 1068)

	Pre-COVID-19 (n = 550)	COVID-19 (n = 518)
	n (%)	n (%)
Age	53.01 ± 0.51	49.57 ± 0.52
Gender		
Male	193 (35.1)	196 (37.8)
Female	357 (64.9)	322 (62.2)
Educational level		
Low	31 (5.6)	38 (7.3)
High	519 (94.4)	480 (92.7)
Attendance		
Yes	459 (83.4)	364 (70.3)
No	91 (16.6)	154 (29.7)
Number of days between visits	34.96 ± 1.49	42.71 ± 2.30

Low educational level: < 12 years, high school or lower

High educational level: ≧ 12 years, university and post-graduation or higher

The “n” means the number of visits. A total of 261 people were seen during the Pre-COVID period, and the number of visits was 550. A total of 254 people were seen during the COVID period, with the number of visits was 518

**Table 2:** Patient characteristics and their dental visit in Pre-COVID-19 and During COVID-19 periods

	Pre-COVID-19			COVID-19		
	Attendance	Non-attendance	p value	Attendance	Non-attendance	p value
	n (%)	n (%)		n (%)	n (%)	
Number of patient	459 (83.4)	91 (16.6)	< 0.001*	364 (70.3)	154 (29.7)	< 0.001*
Age	52.93 ± 0.54	53.40 ± 1.52	0.74	49.27 ± 0.58	50.27 ± 0.52	0.37
Gender						
Male	160 (34.9)	33 (36.3)	0.80	144 (39.6)	52 (33.8)	0.21
Female	299 (65.1)	58 (63.7)		220 (60.4)	102 (66.2)	
Educational level			< 0.001*			0.53
Low	16 (3.5)	15 (16.5)		25 (6.9)	13 (8.4)	
High	443 (96.5)	76 (83.5)		339 (93.1)	141 (91.6)	

p-value was analyzed by Chi-square test (gender and educational level) and two-sample t-test (number of patient and age)

*p < 0.05

During the pre-COVID-19 period, patient with a low education level (< 12 years) accounted for 16.5% of the patients who missed their appointed treatment, and those with a high educational level (≧12 years) accounted for the other 83.5%. During the COVID-19 period, patients with low and high educational levels accounted for 8.4% and 91.6%, respectively. During the pandemic, the proportion of patients with high education levels who did not attend the dental appointment was higher than before the pandemic. During the COVID-19 period, the distribution of educational levels in patients who attended and missed appointments were similar, but in the pre-COVID-19 period, a statistically significant difference was observed (χ^2^ = 24.122, p < 0.0001). The proportion of patients with low educational level who attended their appointments during the pre-COVID-19 and COVID-19 period were 3.5% and 6.9%, respectively. The proportion of those with a high educational level who attended return appointments decreased significantly from 96.5 to 93.1% during the pandemic (χ^2^ = 4.906, p = 0.027). The proportion of patients with a high educational level who missed their appointments during COVID-19 (91.6%) was higher than that during the pre-COVID-19(83.5%). In the analysis of the risk factors for non-attendance (Table 3), patient non-attendance during COVID were 2.09 times higher than pre-COVID after adjusting other factors (p < 0.0001). In the overall analysis, highly educated patients had significantly lower risk of dental visits (adjusted OR = 0.41, p = 0.0007). Further analysis the dental visits during COVID and during pre-COVID was found that high education patients still had significantly higher visit risk than low education patients during pre-COVID (p < 0.0001). But educational level did not make a difference in dental visit during COVID (Table 3).

**Table 3:** The factors for non-attendance by multivariable logistic regression analysis

Independent variables	Risk factors for non-attendance								
				Pre-COVID			COVID		
	Adjusted OR	95% CI	p-value	Adjusted OR	95% CI	p-value	Adjusted OR	95% CI	p-value
Period: COVID (reference: pre-COVID)	2.09	1.55–2.82	< 0.0001*	–	–	–	–	–	–
Age: ≧ 50 yrs (reference: < 50 yrs)	0.89	0.66–1.21	0.466	0.64	0.39–1.04	0.072	1.12	0.76–1.65	0.563
Sex: men (reference: women)	0.92	0.68–1.25	0.601	1.21	0.74–1.97	0.439	0.78	0.52–1.15	0.215
Education level: high (reference: low)	0.41	0.24–0.69	0.0007*	0.16	0.07–0.34	< 0.0001*	0.86	0.43–1.81	0.680

OR: odds ratio

CI: confidence interval

yrs: years old

Adjusted factors: period, age, gender, education level

p-value was analyzed by multiple logistic regression

*p < 0.05

**Fig. 1:**
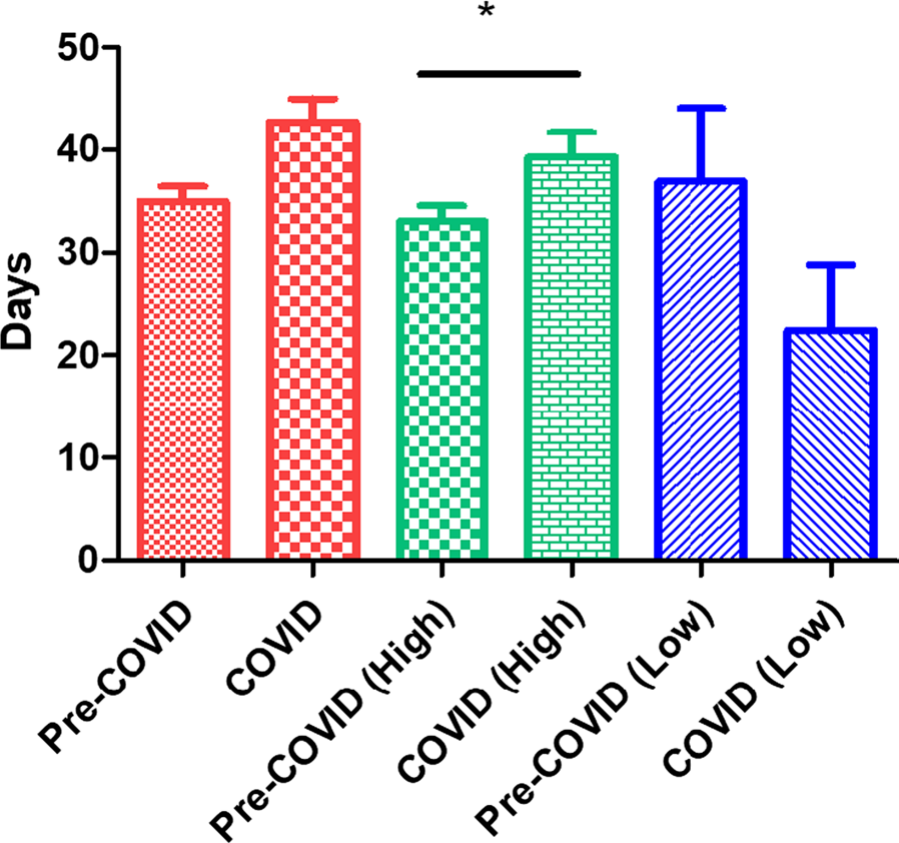
Number of days between visit in Pre-COVID-19 and During COVID-19 period. *p < 0.05

We analyzed the numbers of days between initial visits and return visits by gender and educational level, as presented in Table 4 and Fig. 1. During the COVID-19 period, patients with a high educational level had significantly longer intervals between two appointments than did those with a low educational level in During COVID-19 period (p = 0.019). However, the educational level was unrelated to the length of appointment intervals during the pre-COVID-19 period. No significant difference in the distribution of gender correlated with the length of appointment interval was noted in either study period.

**Table 4:** Appointment interval, gender and educational level during Pre-COVID-19 and During COVID-19 periods

	Pre-COVID-19	COVID-19
	Number of days between visits	Number of days between visits
All patients	34.96 ± 1.49	42.71 ± 2.30
All attendance patients		
Educational level		
Low	36.88 ± 7.22	22.46 ± 6.34
High	33.00 ± 1.58	39.26 ± 2.47
p value	0.61	0.02*
Gender		
Male	33.65 ± 2.51	36.04 ± 3.92
Female	32.86 ± 1.95	39.42 ± 2.94
p value	0.81	0.49

Number of days between visits in Pre-COVID-19: number of days between the return visit and previous visit

Number of days between visits in During COVID-19: number of days between the return visit and the next appointment

*p < 0.05 using two-sample t-test

## Discussion

Dental treatment often causes the dispersion of moisture and aerosols. During the pandemic of COVID-19, many patients had concerns about dental treatment because of the risk of infection. Dental treatments and follow-up visits are often not urgent and can be spread out over a long period. General dental treatment in Taiwan is largely by appointment, with patients often scheduled to return to the clinic for further treatment. Another study revealed a significant decrease in the number of dental patients admitted weekly in Poland before and during the COVID-19 pandemic [12]. During the pandemic, patients’ willingness to visit hospitals or dental clinics for nonurgent dental treatments was decreased [13]. This literature has focused the effects of COVID-19 pandemic on habit change of emergency dental service, but rare studies provide this effect on routine dental services. When patients cancel appointments, other patients typically make appointments to fill the vacated time slots. Although the overall number of appointments declined when the threat in fears of COVID-19 infection rate grew, the decrease was not significant. This finding differs from those of studies on emergency dental procedure during COVID-19 [13].

Although the number of appointments during the COVID-19 and pre-COVID-19 periods was similar, the rate of missed appointments was significantly higher during COVID-19 period. The missed appointment rates were 29.7% and 16.6% (p < 0.001) during the in COVID-19 and pre-COVID-19 periods, respectively. A Brazilian study in 2020 surveyed 595 participants and reported that only 38.3% of them would attend an initial appointment, 44.2% would make a return visit only if they had urgent dental problems, and 17.5% would not return to the dentist for any reason during the COVID-19 pandemic [6]. According to a questionnaire study in 2020 in Madrid, Spain [14], 43.7% of respondents would not make return visits to the dental clinic during the pandemic; 24.5% for fear of the spread of COVID-19 and 16% for economic considerations. Both studies indicated missed appointment rates are higher than those in our study. Possible reasons for this discrepancy include the different survey method, with most other studies using questionnaires. Because our study data was extracted from the actual outpatient records, our findings are more reflective of the actual situation. Second, the severity of the pandemic and public policies differ globally and may influence patient attitudes toward the return visit of dental treatment.

In our research, the average age of patients during the COVID-19 period was less than that during the pre-COVID-19 period. Other studies have indicated that older patients have more physical comorbidities and are more anxious about COVID-19 transmission [15]. This may be the reason why the mean age of participants in the COVID-19 period was significantly lower than that in the pre-COVID period.

Many studies have reported that women’s attitudes towards oral hygiene and medical treatment are generally positive [16–18]. However, one study [6] discovered that men were calmer than women in the face of the pandemic. In our research, no significant difference was noted in the return rate or interval between visits. In this study, we have more women (210 women compared to 122 men), it may be reflected the women in Taiwan were more prone to look for dental procedures. Previous studies showed that females had better attitudes and behavior than males [16]. Women utilized more health services than men [19] and there was a significantly higher number of females that attended dental appointments in compared to males [20]. These situations are similar with ours. In our study, women utilized more dental medical resources than men, both before and during the pandemic. However, compared with men, female patients do not have a higher degree of participation in all medical care. In drug-related medical compliance studies, the data show that male patients have higher drug compliance than women [21].

The findings of this study reveal that the educational level affects the likelihood of patient's making a return dental visit. During the COVID-19 period, the proportion of patients with high educational level who returned to receive treatment was reduced, and their intervals between two appointments were longer. This indicated that highly educated patients might concern more about the safety of attending dental appointment during the pandemic. In other studies on education and medical attitudes, low-level medical attitudes have been associated with anxiety [22–24], but few studies mentioned the effects of education and attitudes toward the general treatment of dentistry. Regarding the correlation between education level and dental visit attendance, a study in 2021 pointed out that the higher education level has 2.19–4.40 times the dental visit attendance compared with the lower education level [11]. One study reported that more than two-thirds of medical students were anxious about COVID-19, and the proportion was even higher among postgraduate students [25]. Although this situation may not necessarily directly determine the number of patients attending dental clinics, it may indirectly explain our finding that people with high educational levels are more concerned about the threat of COVID-19 infection.

Our study has some limitations. Because of the small sample size, we did not classify the types of treatments. The interval between two visits for different dental treatments varies greatly. Besides, the sample of low educational level patients is too low. This may affect the result of our analysis. In the future, we must divide the dataset by the different dental treatment types and analyze them separately and increase the number of cases. Due to the ever-changing epidemic situation, the research time interval of the two months may be too short. However, this can also reflect the impact of the epidemic on dental care in a timely manner. If the epidemic continues to spread for a long time, a longer research interval should be invested to more truly reflect the real situation. Second, data from the outpatient clinics can be used to determine whether a patient actually seeks medical attention, an advantage over using questionnaires or telephone interviews; however, it did not allow us to learn the reasons of patients attended and not to attend their appointments. Third, our sample were in a medical center it may have been influenced the results and could not reflect the situation in regional hospitals or local clinics.

Our findings provide insight into patient attitudes toward dental visits during pandemic periods. In addition to revealing patient anxiety toward dental care during the pandemic, this study provides a reference for the arrangement of patient appointments under pandemic conditions.

## Conclusion

During the COVID-19 pandemic, the attendance rate for return dental treatment appointment declined, especially in high educational level. Confirming a patient’s intent to attend prior to the appointment, especially for highly educated patients, may enable dental clinic resources to be used more efficiently.

## Data Availability

The datasets generated and/or analysed during the current study are not publicly available due patients' privacy but are available from the corresponding author on reasonable request.

## Abbreviation

COVID-19: Coronavirus disease 2019
